# A novel C-type lectin from *Trichinella spiralis* mediates larval invasion of host intestinal epithelial cells

**DOI:** 10.1186/s13567-022-01104-2

**Published:** 2022-10-18

**Authors:** Hui Nan Hao, Yan Yan Song, Kai Ning Ma, Bo Ning Wang, Shao Rong Long, Ruo Dan Liu, Xi Zhang, Zhong Quan Wang, Jing Cui

**Affiliations:** Department of Parasitology, Medical College, Zhengzhou University, Zhengzhou, 450052 China

**Keywords:** *Trichinella spiralis*, C-type lectin; intestinal epithelial cells (IEC), invasion, mannose

## Abstract

The aim of this study was to investigate the characteristics of a novel type C lectin from *Trichinella spiralis* (TsCTL) and its role in larval invasion of intestinal epithelial cells (IECs). TsCTL has a carbohydrate recognition domain (CRD) of C-type lectin. The full-length TsCTL cDNA sequence was cloned and expressed in *Escherichia coli* BL21. The results of qPCR, Western blotting and immunofluorescence assays (IFAs) showed that TsCTL was a surface and secretory protein that was highly expressed at the *T. spiralis* intestinal infective larva (IIL) stages and primarily located at the cuticle, stichosome and embryos of the parasite. rTsCTL could specifically bind with IECs, and the binding site was localized in the IEC nucleus and cytoplasm. The IFA results showed that natural TsCTL was secreted and bound to the enteral epithelium at the intestinal stage of *T. spiralis* infection. The rTsCTL had a haemagglutinating effect on murine erythrocytes, while mannose was able to inhibit the rTsCTL agglutinating effect for mouse erythrocytes. rTsCTL accelerated larval intrusion into the IECs, whereas anti-rTsCTL antibodies and mannose significantly impeded larval intrusion in a dose-dependent manner. The results indicated that TsCTL specifically binds to IECs and promotes larval invasion of intestinal epithelium, and it might be a potential target of vaccines against *T. spiralis* enteral stages*.*

## Introduction

Trichinellosis is a worldwide foodborne parasitic disease that results from the ingestion of raw or poorly cooked meat containing *Trichinella* muscle larvae (MLs) [1]. Mammals, rodents, amphibians, reptiles and birds can act as *Trichinella* hosts. *Trichinella* infection in humans is mainly caused by eating infected swine pork, horse or wild animal meat [2–4]. In Argentina and Chile, 6662 and 258 trichinellosis patients were documented in 2012–2020 and 2005–2015, respectively [5]. In China, eight outbreaks of human trichinellosis with 479 cases and 2 deaths were reported from 2009 to 2020, and the endemic area of trichinellosis is principally located in the southwestern regions of China. Seven out of the 8 outbreaks (87.50%) involved eating raw or semicooked pork [6]. Pork from domestic pigs remains the main source of trichinellosis outbreaks. *Trichinella* infection has become a major foodborne zoonosis that is not only an important public health issue but also a threat to animal food safety [7]. However, it is difficult to eliminate *Trichinella* infection in animals because of its broad host range and lack of available anti-*Trichinella* vaccines [8, 9]. Therefore, development of preventive vaccines is needed to block the transmission of porcine *Trichinella* infection and eliminate the infective larvae in pork and pork products [10].

After ingestion, *T. spiralis* MLs are released from the collagen capsules under the action of host gastric fluid. MLs are activated into intestinal infectious larvae (IILs) by gut contents or bile [11]. IILs penetrate intestinal epithelial cells (IECs) and develop into adult worms (AWs) after molting 4 times. After copulation, the pregnant female AW produces newborn larvae (NBLs), which enter the lymphatic and blood circulation and penetrate into skeletal muscles to develop into the encapsulated MLs to finish the lifecycle [12]. The IIL intrusion of IECs is the first pivotal step for intestinal *T. spiralis* infection. The gut epithelium is the first native physical barrier against *T. spiralis* intrusion and the prime interaction location of the parasite and host [13, 14], but the mechanism of IIL intrusion of the IECs is not clear [15, 16]. Characterization of IIL intrusion molecules will be valuable to elucidate the IIL intrusion mechanism and to develop preventive vaccines for interdicting *Trichinella* invasion of gut mucosa [17, 18].

Lectin is a kind of protein that agglutinates red blood cells, lymphocytes, fibroblasts, etc., and binds reversibly to carbohydrates present on the cells. Lectin has the carbohydrate recognition domain (CRD), which binds directly to the surfaces of host cells, and the binding of lectin with the ligand on host cells can be inhibited by one or more carbohydrates. The CRD exhibits specificity towards oligosaccharides, including mannose, N-acetylglucosamine and fucose residues [19]. According to the molecular structure, animal lectin is divided into several types: C (including selectin), S (galectin), P and I types. C-type lectin (CTL) is a superfamily of more than 1000 proteins with one or more type C lectin domains, which generally contains 110–130 amino acids, and it has a typical double loop structure [20]. The striking feature of lectin is agglutinating erythrocytes; lectin has at least 2 carbohydrate-binding sites that can bind to glycoproteins on the erythrocyte surface, leading to cell crosslinking and precipitation. In addition to sugars, CTLs can also recognize a variety of ligands, such as lipids, proteins, and uric acid crystals [21]. Lectins from hosts have been well studied but less studied in parasitic nematodes [22]. Previous studies have shown that parasite-derived lectins are mainly involved in parasite adhesion and invasion to host cells [23, 24]. Nevertheless, there are no reports in the literature on the biological properties and function of worm-derived CTLs during *T. spiralis* infection.

In this study, a novel CTL domain protein from the parasitic nematode *T. spiralis* (TsCTL, GenBank: KRY42391.1) was obtained from the *T. spiralis* draft genome [25]. TsCTL has a CRD of the C-type lectin. The aim of the current study was to assess the biological properties of TsCTL and its role in *T. spiralis* invasion of host intestinal mucosa.

## Materials and methods

### Parasites, experimental animals and cells

A *Trichinella spiralis* isolate (ISS534) was isolated from a naturally infected pig in Henan Province, China [26]. The isolate was passaged in BALB/c mice in our laboratory every 6 months. Female 6- to 8-week-old BALB/c mice were purchased from the Henan Provincial Experimental Animal Center (No. SCXK 2020–0004). These mice were bred in individual ventilated cages (IVC, Suzhou Fengshi Laboratory Animal Equipment Co., Ltd., Suzhou, China) [27]. Intestinal epithelium cells (IECs) were isolated from the small intestine of normal BALB/c mice, and negative control C2C12 cells were from mouse skeletal muscle myoblast cells that are insensitive to *Trichinella* larval penetration [28, 29].

### Worm collection and protein preparation

MLs were obtained by artificially digesting *T. spiralis-*infected mouse muscles at 42 days post-infection (dpi). The IILs and AWs were recovered from the small intestine of infected mice at 6 h post-infection (hpi) and 2, 3 and 6 dpi, respectively [30, 31]. Six-day-old female adults were cultured in RPMI-1640 with 10% foetal bovine serum (FBS; Gibco, New Zealand) at 37 °C in 5% CO_2_ for 24 h, and the NBLs were harvested [32]. Soluble worm somatic proteins of various stage worms (MLs, IILs, AWs and NBLs), excretory/secretory antigens (ESA) from MLs, IILs and 6 d AWs were prepared as described previously [33]. Briefly, the diverse stage worms were first homogenized with a tissue grinder (KZ-II Servicebio), and worm tissue fragments were further homogenized using ultrasonication. The supernatant carrying worm soluble proteins was obtained after centrifugation at 15 000 × *g* for 1 h at 4 °C. Moreover, to prepare the ESA, the worms were washed using sterile saline and cultured in RPMI-1640 medium (5000 worms/mL) at 37 °C and 5% CO_2_ for 18 h. The culture medium containing ESA was filtered through a 0.22 μm membrane and concentrated using an ultrafiltration tube. The concentration of soluble proteins and ESA was ascertained by a Coomassie brilliant blue G-250 method [17].

### Bioinformatics analysis and evolutionary tree construction

The full-length cDNA sequence of the TsCTL gene (GenBank: KRY42391.1) was obtained from NCBI. Bioinformatics analysis software (SignalP, TargetP, SMART, ProtParam, DNAstar, Swiss-Model) was used to analyse and predict its physicochemical properties, such as the signal peptide, subcellular localization, tertiary structure and functional sites [29]. The amino acid sequences of TsCTL were compared with those of C-type lectins from other organisms with BioEdit software. The GenBank accession numbers of other organism C-type lectins were as follows: *T. nativa* (KRZ61993.1), *Trichinella* T9 (KRX55578.1), *Trichinella* T8 (KRZ84598.1), *T. murrelli* (KRX40013.1), *T. britovi* (KRY48405.1), *T. patagoniensis* (KRY12567.1), *Trichinella* T6 (KRX75278.1), *T. nelsoni* (KRX18392.1), *T. pseudospiralis* (KRY00679.1), *T. zimbabwensis* (KRZ11314.1), and *T. papuae* (KRZ71253.1). *Trichuris suis* (KFD49507.1), *Trichuris trichiura* (CDW53534.1), *Mus musculus* (AAD05125.1) and *Homo sapiens* (KAI2580953.1). The multisequence alignment and phylogenetic tree analysis of TsCTL were performed with the neighbour-joining (NJ) method by using Jalview and MEGA 7.0 [28, 34].

### Cloning, expression and identification of rTsCTL

Total RNA was obtained from *T. spiralis* IIL with TRIzol reagent (Invitrogen, USA) and reverse transcribed into cDNA. The complete TsCTL cDNA sequence was amplified using PCR by specific primers with *BamHI* and *SalI* restriction enzyme sites (bold). The specific primers were 5′-C**GGATCC**AACCGTTTTCCGTGCCGTATCAAAT-3′ and 5′-ACGC**GTCGAC**TCACTCCAACGAATGACAAATTC-3′. The PCR products were cloned into pQE-80L with an N-terminal His-tag, and the recombinant plasmid pQE-80L/TsCTL was transferred into *Escherichia coli* BL21 (DE3) (Novagen, USA). After being induced with 0.4 mM IPTG at 25 °C for 8 h, rTsCTL was expressed, purified using a Ni–NTA His-tag affinity kit (Novagen) [35], and identified by SDS‒PAGE and Western blot as previously described [15].

### Preparation of anti-rTsCTL serum

Forty mice were immunized subcutaneously with 20 μg rTsCTL protein emulsified with complete Freund’s adjuvant and boosted three times by 20 μg rTsCTL emulsified with incomplete Freund’s adjuvant at a two-week interval [36]. At 2 weeks after the fourth immunization, the tail blood from immunized mice was collected to isolate anti-rTsCTL sera; preimmune sera were also collected to be used as a negative control [37, 38].

### Western blot analysis

Soluble crude and ES proteins of different *T. spiralis* stages and purified rTsCTL were separated by 10% SDS‒PAGE [39, 40]. The proteins were transferred onto a nitrocellulose (NC) membrane (Millipore, USA) in a semidry transfer cell (Bio-Rad, USA) [41, 42]. The membrane was blocked with 5% skim milk in Tris-buffered saline containing 0.05% Tween (TBST) at 37 °C for 2 h and incised into strips. The strips were probed with various sera (1:100; anti-rTsCTL serum, infection serum and preimmune serum) at 37 °C for 2 h. After washes with TBST, the strips were incubated at 37 °C for 1 h with HRP-anti-mouse IgG conjugate (1:10 000; Southern Biotech, USA). After being washed again, the strips were coloured using 3,3’-diaminobenzidine tetrahydrochloride (DAB; Sigma-Aldrich, USA) [9, 43].

### qPCR assay

Total RNA from various *T. spiralis* phases (MLs, IILs, 3 d AWs, and NBLs) was isolated using TRIzol reagent (Invitrogen). The TsCTL mRNA transcription level at diverse worm stages was ascertained by qPCR as described previously [44, 45]. TsCTL-specific primers for qPCR were 5'-AACAAAATCGAATGCCGAAG-3' and 5'-TAGTCACAATTCCACTCGCTT-3'. The relative TsCTL mRNA expression level was normalized by subtracting the mRNA expression level of the *T. spiralis* housekeeping gene GAPDH (GenBank: AF452239) [46] and then calculated on the basis of the comparative Ct (^2−ΔΔCt^) method [47, 48]. Each experiment had three replicates.

### Immunofluorescence assay (IFA)

IFA was performed to determine the tissue location of the TsCTL protein in *T. spiralis* worms as reported previously [49, 50]. Whole worms of various *T. spiralis* stages (MLs, IILs, AWs and NBLs) were fixed with 4% paraformaldehyde and embedded in paraffin, and 2-µm-thick worm cross-sections were cut with a microtome [41]. Whole *T. spiralis* and cross-sections were blocked with 5% goat serum at 37 °C for 1 h, washed three times in PBS, and then incubated with 1:10 dilutions of anti-rTsCTL serum, infection serum and preimmune serum. Goat anti-mouse IgG-FITC conjugate (1:100; Abways, Shanghai, China) was used as the secondary antibody. After more washes, the whole worms and cross-sections were observed under fluorescence microscopy (Olympus, Japan) [37, 51].

### Far-Western blot analysis

The binding of rTsCTL and IEC was investigated by far-Western blot analysis as previously described [16]. In brief, soluble IEC proteins were first separated by SDS‒PAGE and then transferred to NC membranes (Millipore, USA). The membrane was cut into strips, blocked with 5% skim milk at 37 °C for 2 h, and then incubated with 20 μg/mL rTsCTL for 2 h at 37 °C. Following washes with PBST, strips were probed at 37 °C for 1 h with anti-rTsCTL serum (1:100) as the primary antibody and HRP-anti-mouse IgG (1:10 000; Southern Biotech) as the secondary antibody [52]. After washing, colouration was developed using 3-amino-9-ethylcarbazole (AEC, Solarbio, China), and the protein bands were analysed by AlphaView software [18, 53].

### IFA analysis of the binding of rTsCTL and IECs

The binding of rTsCTL and IECs and its cellular localization were also assessed using IFA [39]. IECs were cultivated in a 6-well culture plate until confluence [11]. IECs were incubated with rTsCTL (20 μg/mL) at 37 °C for 2 h. After being washed with PBS, IECs were fixed with 4% paraformaldehyde for 10 min and subsequently blocked with 5% goat serum at 37 °C for 2 h. IECs were incubated with anti-rTsCTL serum (1:10). FITC-anti-mouse IgG-conjugate (1:100; Abways, Shanghai, China) was used as the secondary antibody. Cell nuclei were stained blue with 4',6-diamidino-2-phenylindole (DAPI, Solarbio), and the cells were observed by fluorescence microscopy and an Olympus FV1200 laser scanning microscope [54]. Images were captured by using an Olympus FV1200 laser scanning microscope and analysed by using Olympus Fluoview software [55].

### IFA analysis of TsCTL binding with normal murine gut epithelium

For determination of the ability of TsCTL to bind normal murine gut epithelium, small intestines from normal mice were fixed with paraformaldehyde, 3-μm intestinal tissue sections were prepared, and IFA was performed as described previously [56]. Briefly, after the sections were blocked using 5% goat serum, they were incubated with rTsCTL or IIL ESA (20 μg/mL) for 1 h at 37 ℃, and the subsequent procedures were the same as those used for IFA.

For determination of whether natural TsCTL could be secreted and bound to gut epithelium, eight mice were infected orally with 3000 MLs. Two infected mice were sacrificed at 1, 3, 7 and 14 dpi, and the intestine was also collected to prepare intestinal sections for IFA analysis [57].

### Haemagglutination activity and sugar inhibition assay

Mouse erythrocytes were collected and suspended in 2% TBS buffer (200 mM Tris–HCl, 150 mM NaCl, pH 8.0). Different concentrations of rTsCTL (0–400 μg/mL) were added to TBS buffer. After being mixed, the mixture was added to TBS buffer containing 10 mM CaCl_2_. Bovine serum albumin (BSA, Sigma-Aldrich, USA) was used as an irrelevant protein control, and PBS was used as a negative control. The plates were incubated for 1 h at room temperature, complete agglutination was observed by the naked eye, and the lowest dose of induced erythrocyte agglutination was recorded [58]. The experiment was performed in triplicate.

A sugar inhibition assay was performed according to previous studies [59]. Four different carbohydrates, lactose, sucrose, glucose and mannose, were used in this assay. rTsCTL (100 μg/mL) was added to the U-shaped plate, and 25 μL of different dilutions (100–400 mM) of carbohydrates were subsequently added to the well. After being incubated for 1 h at room temperature, 2% suspensions of mouse erythrocytes were added and incubated at room temperature for another 1 h to observe haemagglutination inhibition. Two control assays were performed without rTsCTL or carbohydrate, which was replaced with PBS.

### The in vitro larval invasion test

For analysis of the accelerative role of TsCTL during larval invasion of the gut epithelium, an in vitro invasion test was conducted as previously reported [60]. Briefly, MLs were activated into IILs with 5% porcine bile at 37 °C for 2 h, and different doses of rTsCTL (0–15 μg/mL) and one hundred IILs were added to semisolid medium. Moreover, the same concentration of BSA was used as a control. After culture at 5% CO_2_ at 37 °C for 2 h, larval intrusion of IECs was examined by microscopy. The IILs invading IECs were active and migrated within the cell monolayer, while the noninvaded IILs coiled on the cell monolayer surface [61, 62]. In the haemagglutination activity assay, only mannose of the 4 carbohydrates had an inhibitory effect on the agglutination of mouse erythrocytes by rTsCTL. Therefore, for further analysis of the suppressive function of mannose on larval invasion, mannose was used in the in vitro larval invasion test. IILs were first incubated with different doses (0–400 mM) of mannose at 37 ℃ for 2 h and then added to semisolid medium. After culture at 5% CO_2_ at 37 °C for 2 h, larval penetration into IECs was examined by microscopy. For further analysis of whether mannose could inhibit rTsCTL facilitative on larval invasion of IEC, 10 μg/mL of rTsCTL was first incubated with various doses (0–400 mM) of mannose for 2 h, and the mixture containing rTsCTL, mannose and IILs was added onto the cell monolayer. After culture at 5% CO_2_ at 37 °C for 2 h, larval intrusion of IECs was examined under microscopy [63, 64].

### Statistical analysis

All the data were analysed by SPSS 21.0 software, and the results are shown as the mean ± standard deviation (SD). One-way ANOVA was used to analyse the difference in relative TsCTL mRNA expression levels in various stages. The chi square test was used to compare the differences in larval invasion among the different groups. The correlation between the doses of rTsCTL, anti-rTsCTL antibodies and mannose and larval invasion was analysed by linear regression. *P* < 0.05 was defined as statistically significant.

## Results

### Bioinformatics analysis of TsCTL

**Figure 1:**
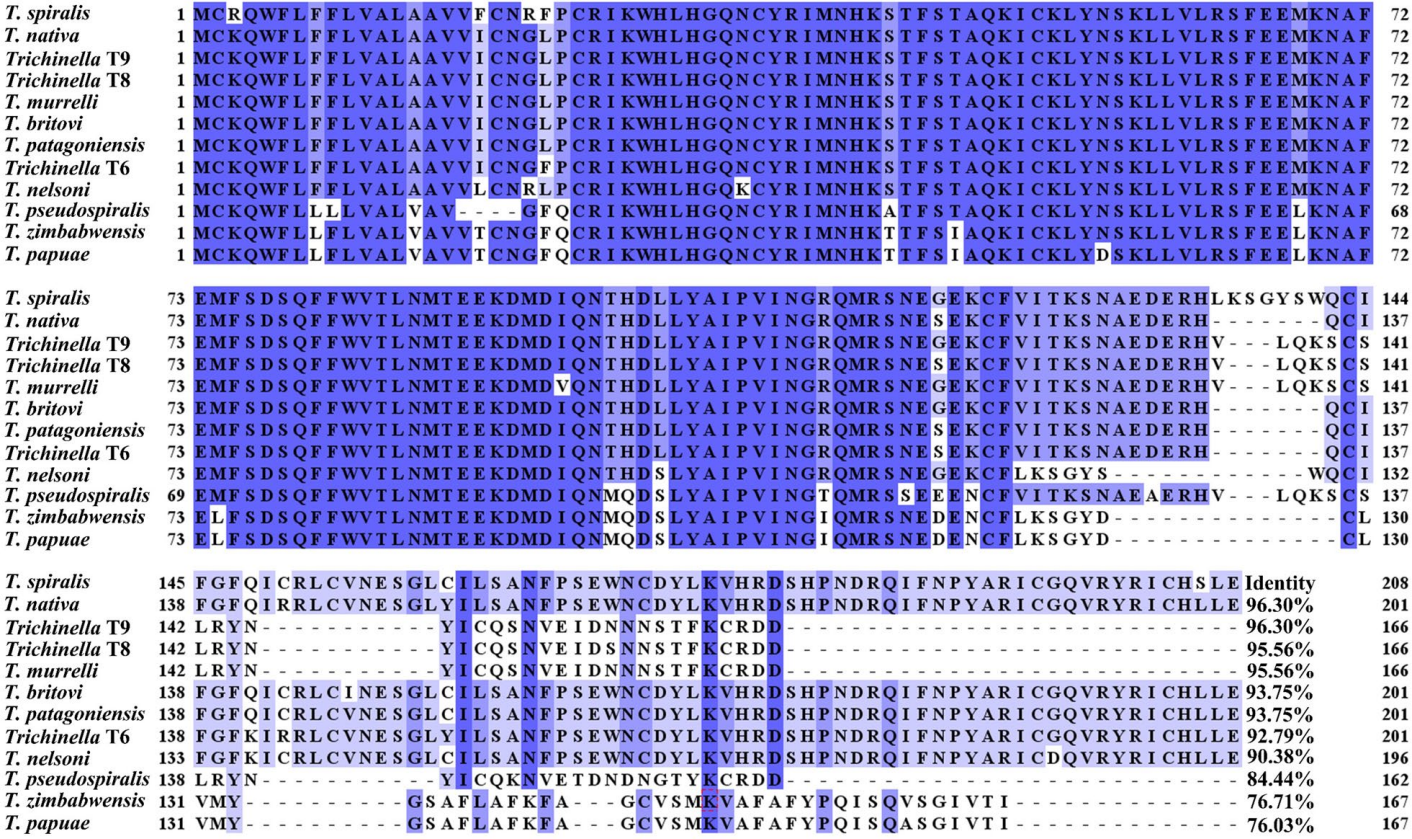
**Sequence alignment of TsCTL with other** ***Trichinella*** **species or genotypes.** Jalview and MEGA 7.0 were used to analyse the sequences, and distinct differences were observed in various *Trichinella* species/genotypes. Blue shading indicates residues identical to TsCTL, and medium blue shading shows conservative substitution of amino acid residues.

**Figure 2:**
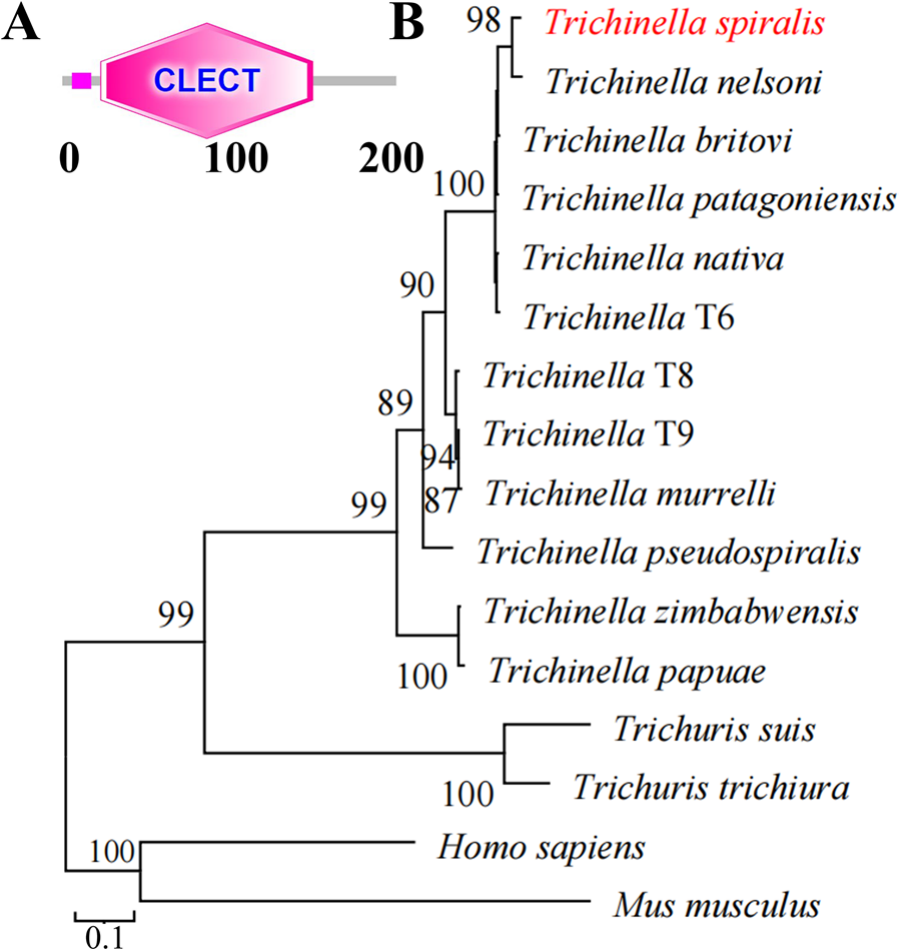
**Predicted structure of TsCTL (A) and phylogenetic tree of C-type lectin of 16 organisms with the NJ method (B). A:** The C-type lectin domain (CLECT) was localized at residues 24–156.

The complete TsCTL cDNA sequence is 627 bp, encoding 208 aa, with a molecular weight of 24 kDa and pI of 8.19. TsCTL contains a signal peptide, has obvious hydrophobicity at the N-terminus, and contains a transmembrane region. Subcellular localization predicted that TsCTL is a secretory protein. The amino acid sequences of TsCTL had an identity of 96.30, 96.30, 95.56, 95.56, 93.75, 93.75, 92.79 and 90.38% with C-type lectins of the 8 encapsulated *Trichinella* species/genotypes (*T. nativa*, *Trichinella* T9, *Trichinella* T8, *T. murrelli*, *T. britovi*, *T. patagoniensis*, *Trichinella* T6, *T. nelsoni*), and it had an identity of 84.44, 76.71 and 76.03% with C-type lectins from 3 nonencapsulated *Trichinella* species (*T. pseudospiralis*, *T. zimbabwensis* and *T. papuae*) (Figure 1). TsCTL structural prediction showed a CRD of C-type lectin at residues 24–156 (Figure 2A). The phylogenetic tree of TsCTL showed that a monophyletic group of the genus *Trichinella* is well supported, which is closely related to whip worms (*Trichuris suis* and *Trichuris trichiura*) (Figure 2B).

### Expression and identification of rTsCTL

**Figure 3:**
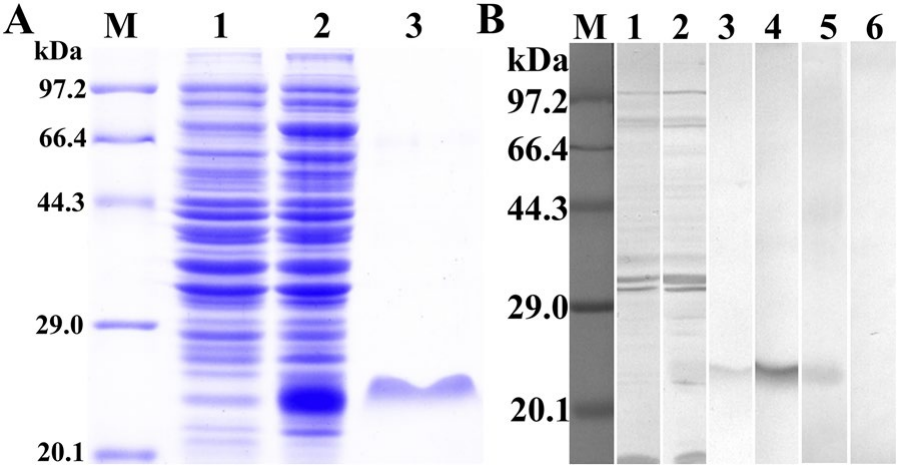
**Expression and antigenicity analyses of rTsCTL. A:** SDS‒PAGE of rTsCTL. Lane M: protein marker; Lane 1: lysate of bacteria carrying pQE-80L/TsCTL prior to induction; Lane 2: lysate of bacteria carrying pQE-80L/TsCTL after induction; Lane 3: purified rTsCTL. **B:** Western blot analysis of rTsCTL. The lysates of bacteria carrying pQE-80L/TsCTL prior to induction (Lane 1) were not recognized by infection serum, and the lysates of induced bacteria carrying pQE-80L/TsCTL (Lane 2) and purified rTsCTL (Lane 3–6) were recognized with infection serum (Lane 3), anti-His monoclonal antibody (Lane 4) and anti-rTsCTL serum (Lane 5) but not by normal serum (Lane 6).

After induction with IPTG, the fusion protein with the His-tag was expressed in *E. coli* BL21 harbouring pQE-80L/TsCTL. The rTsCTL protein was purified using a Ni–NTA-Sepharose column. SDS‒PAGE analysis revealed that rTsCTL had a clearly visible individual band, and its molecular weight (24 kDa) was consistent with its predicted size (Figure 3A). To assess the antibody response elicited by rTsCTL immunization, we determined the titre of anti-rTsCTL IgG at two weeks after four immunizations by ELISAs. The results showed that the IgG titre of anti-rTsCTL antibodies reached 1:10^5^ after four immunizations, indicating that rTsCTL has good antigenicity. Western blot analysis showed that rTsCTL was recognized by anti-rTsCTL immune serum, infection serum, and anti-His-tagged monoclonal antibody (Figure 3B) but not by normal serum.

### Transcription and expression of TsCTL in diverse *T. spiralis* phases

**Figure 4:**
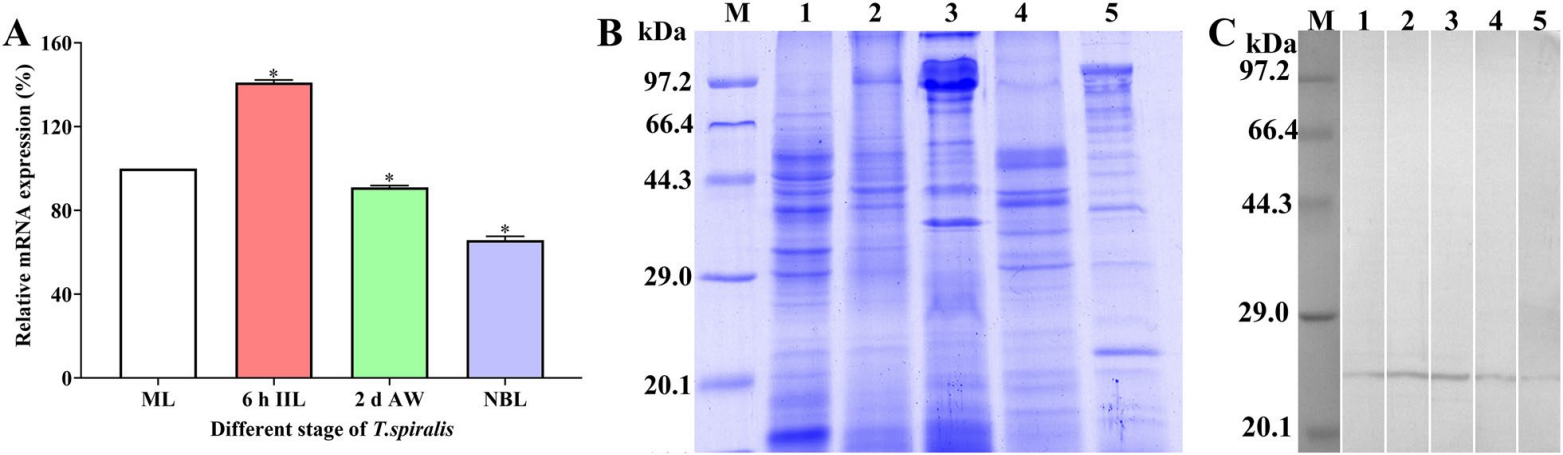
**TsCTL transcription and expression in diverse** ***T. spiralis*** **phases. A:** qPCR assay of TsCTL transcription levels in different worm phases. The relative transcription level of TsCTL at the IIL stage was evidently higher than those of other worm stages, *P < 0.05 compared to the ML stage. **B:** SDS‒PAGE analysis of somatic crude proteins of MLs (Lane 1), 2 h IILs (Lane 2), 6 h IILs (Lane 3), 3 d AWs (Lane 4) and NBLs (Lane 5); Lane M, protein marker. **C:** Western blot analysis of native TsCTL in somatic crude proteins of MLs (Lane 1), 2 h IILs (Lane 2), 6 h IILs (Lane 3), 3 d AWs (Lane 4) and NBLs (Lane 5) identified using anti-rTsCTL serum.

**Figure 5:**
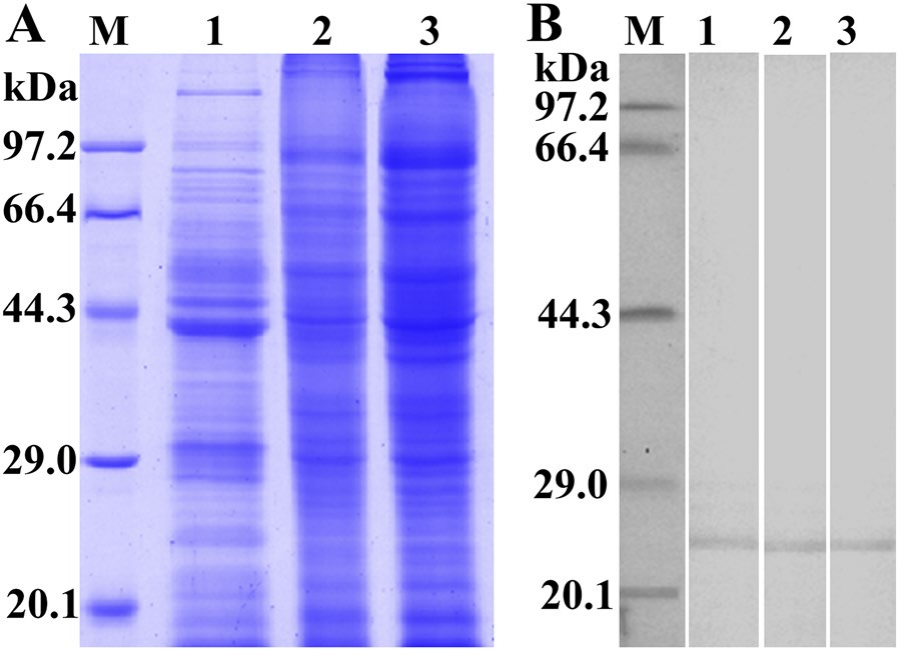
**Western blot identification of native TsCTL in ES proteins of different** ***T. spiralis*** **stages. A:** SDS‒PAGE of ES proteins of different *T. spiralis* stages. Lane M, protein marker; ES proteins of MLs (Lane 1), 6 h IILs (Lane 2) and 6 d AWs (Lane 3). **B:** Western blot analysis of native TsCTL in ES proteins of different worm stages. Native TsCTL in ES proteins of MLs (Lane 1), 6 h IILs (Lane 2) and 6 d AWs (Lane 3) was recognized by anti-rTsCTL serum.

qPCR assays showed that the TsCTL mRNA expression level in the IIL stage was significantly higher than that in the ML stages, but the TsCTL expression level in the 2-day AW and NBL stages was lower than that in the ML stage (*P* < 0.05) (Figure 4A). Somatic soluble proteins of diverse *T. spiralis* stages were isolated by SDS‒PAGE analysis (Figure 4B). Western blot results revealed that native TsCTL in soluble proteins of diverse *T. spiralis* phases (MLs, 2 h IILs, 6 h IILs, 3 day AWs and NBLs) was detected by anti-rTsCTL serum. Native TsCTL protein was identified in worm somatic soluble proteins of diverse *T. spiralis* stages (Figure 4C). Furthermore, natural TsCTL in ES proteins of different worm phases (MLs, 6 h IILs and 6 day AWs) was recognized by anti-rTsCTL serum (Figure 5). The results indicated that TsCTL is a secretory protein of various *T. spiralis* stages, and higher expression of TsCTL in the IIL stage suggested that TsCTL might be an invasion-related protein.

### Expression and worm localization of natural TsCTL in *T. spiralis*

**Figure 6:**
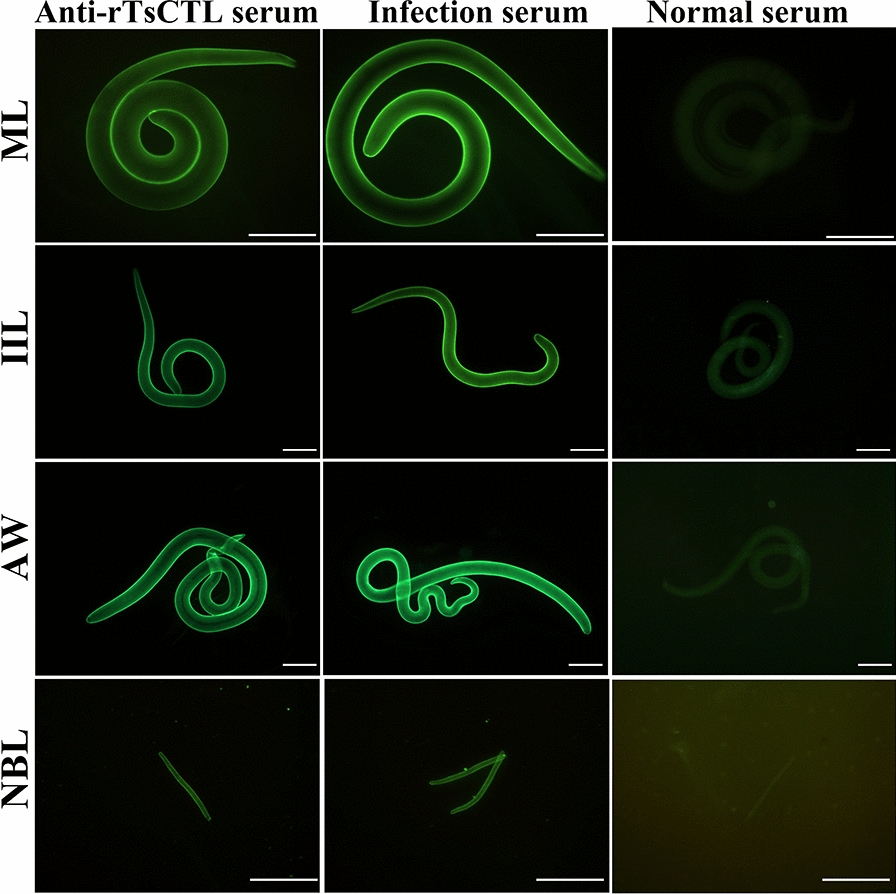
**Expression of TsCTL at the outer cuticle of various** ***T. spiralis*** **stages by IFA.** Whole worms were probed with anti-rTsCTL serum, and immunofluorescence was detected at the epicuticle of ML, IIL, AW and NBL. However, preimmune normal serum did not recognize any worm components of the parasitic nematode. Scale bars = 200 μm.

**Figure 7:**
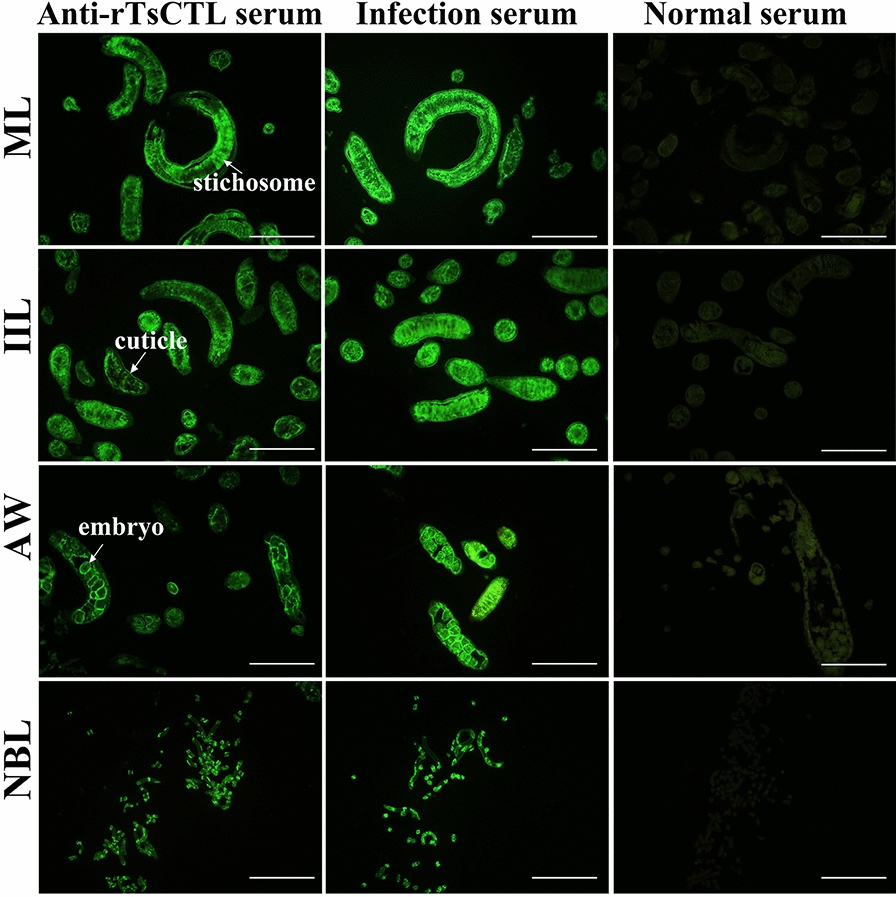
**Immunolocalization of TsCTL in cross-sections of diverse** ***T. spiralis*** **stages by IFA.** Fluorescence staining was observed at the cuticle and stichosome of MLs, IILs, and embryos of the adult females by using anti-rTsCTL serum. No immunostaining in worm cross-sections was observed by using normal serum as a negative control. Scale bars: 200 μm.

The results of IFA with whole parasites revealed green immunofluorescence on the epicuticle of MLs, IILs, AWs and NBLs by using anti-rTsCTL serum and infection serum (Figure 6). When the worm cross-sections were probed by anti-rTsCTL serum, immunostaining was located at the cuticle and stichosome of MLs and IILs and embryos of the female adults (Figure 7). No worm tissue components of the parasites were identified by normal serum.

### Binding of rTsCTL and IEC proteins assessed with far-Western blotting

**Figure 8:**
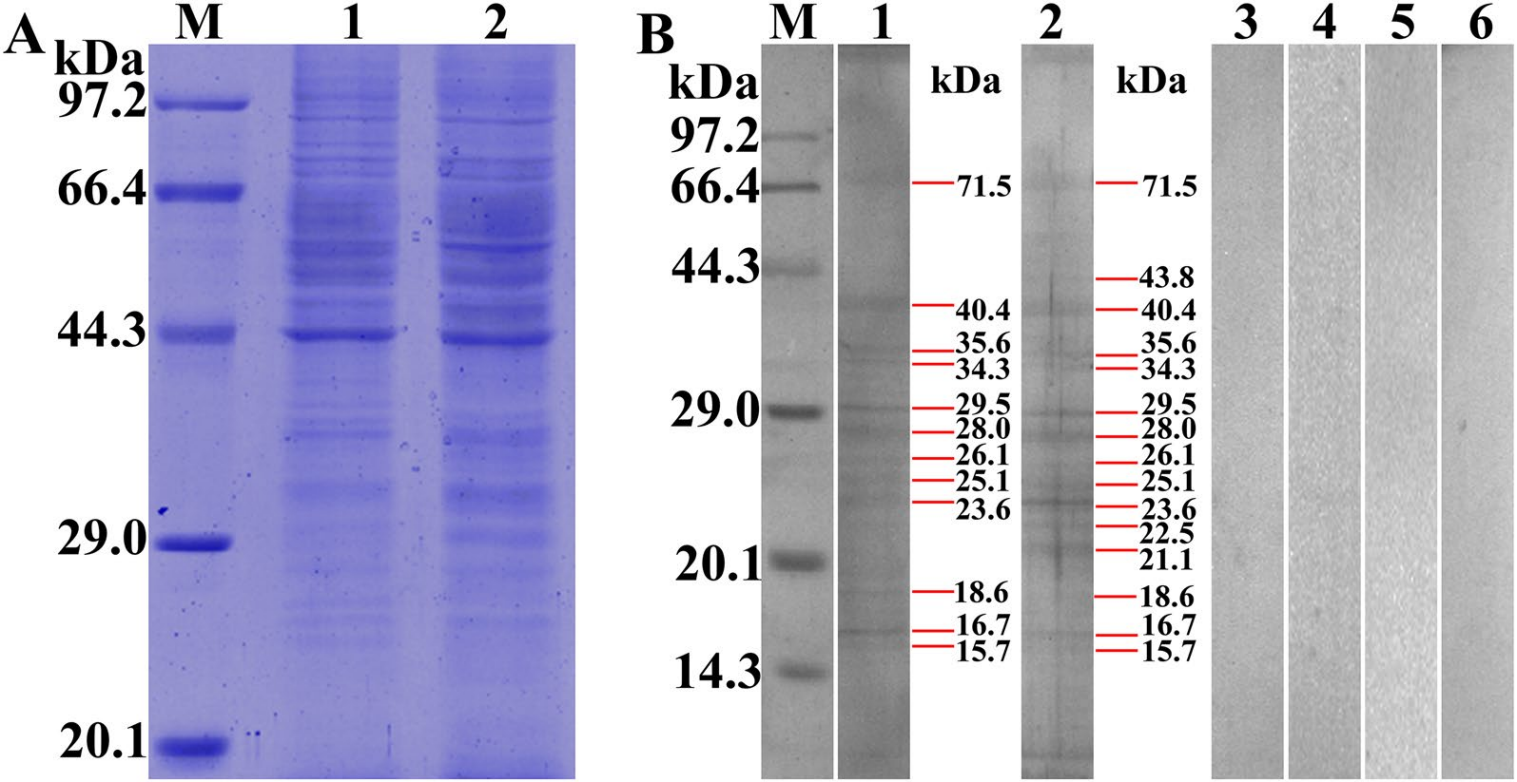
**Far-Western blot identification of binding between rTsCTL and IEC proteins. A:** SDS‒PAGE analysis of soluble IEC proteins. Lane M: protein marker. Lane 1: IEC proteins. Lane 2: C2C12 proteins. **B:** Far-Western blot showing the binding of rTsCTL with IEC proteins. Lane M: protein marker; the strips containing IEC proteins (Lanes 1–3) or C2C12 proteins (Lanes 4–6) were incubated with rTsCTL (Lanes 1–6), and rTsCTL binding with IECs was identified by infection serum (Lane 1) and anti-rTsCTL serum (Lane 2) but not by preimmune serum (Lane 3). No binding of rTsCTL with C2C12s was detected with infection serum (Lane 4), anti-rTsCTL serum (Lane 5) or preimmune serum (Lane 6).

On far-Western blot﻿ analysis, after IEC proteins were incubated with rTsCTL, 12 bands (71.5, 40.4, 35.6, 34.3, 29.5, 28.0, 26.1, 25.1, 23.6, 18.6, 16.7 and 15.7 kDa) were identified with infection serum, and anti-rTsCTL serum identified three more bands (43.8, 22.5 and 21.1 kDa) than infection serum. No IEC proteins preincubated with rTsCTL were identified with preimmune serum, and no C2C12 proteins preincubated with rTsCTL were detected by anti-rTsCTL serum or infection serum (Figure 8). The results indicated that there is a specific binding of TsCTL with IEC proteins.

### Binding of rTsCTL with IECs and its cellular localization

**Figure 9:**
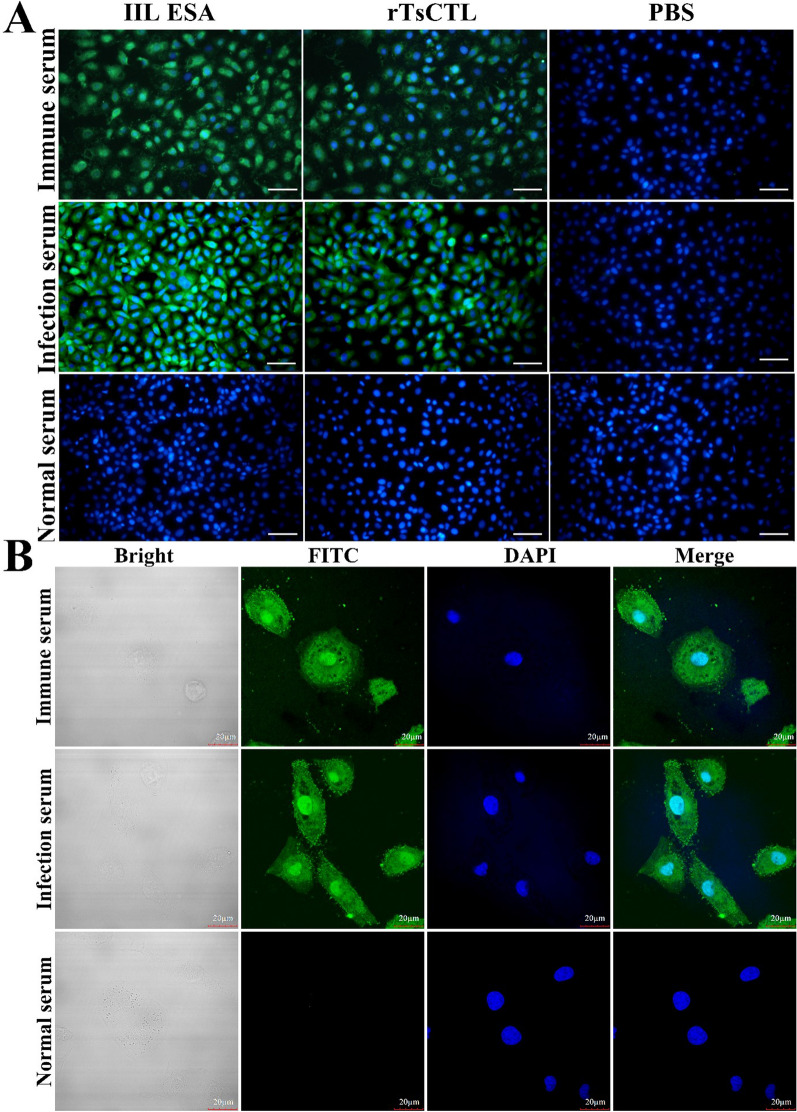
**Specific binding between rTsCTL and IECs detected by IFA. A:** The IECs were preincubated with rTsCTL, IIL ESA or PBS. After blocking and washing, the IECs were probed using anti-rTsCTL serum, infection serum or preimmune serum, followed by incubation with FITC-conjugated anti-mouse IgG. Cell nuclei were redyed blue by DAPI. Scale bars: 200 μm. **B:** Cellular localization of rTsCTL in IECs by confocal microscopy. Scale bars: 20 μm.

The IFA results showed that after IECs were preincubated with rTsCTL, green immunofluorescence staining was observed on the surface of IECs probed with anti-rTsCTL serum and infection serum but not with preimmune serum (Figure 9A). Confocal microscopy showed that immunostaining was mainly localized in the IEC nucleus and cytoplasm (Figure 9B).

### Binding of rTsCTL and native TsCTL with enteral epithelium

**Figure 10:**
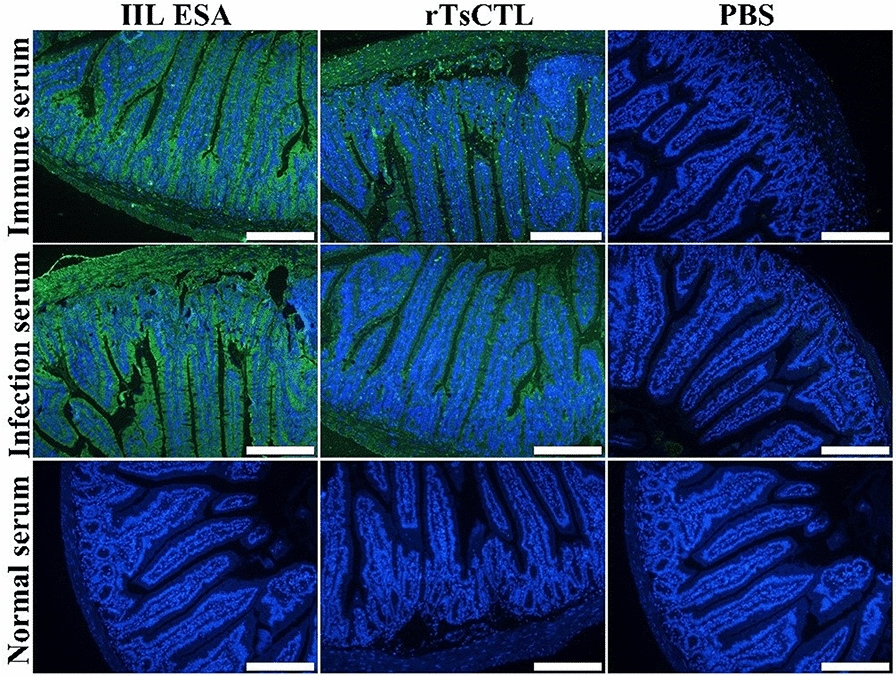
**Binding of rTsCTL with normal murine enteral epithelium.** Intestinal tissue sections from normal mice were incubated with rTsCTL or IIL ESA and then probed with anti-rTsCTL immune serum and infection serum. Intestinal epithelial cell nuclei were coloured blue by DAPI. Scale bars: 200 μm.

**Figure 11:**
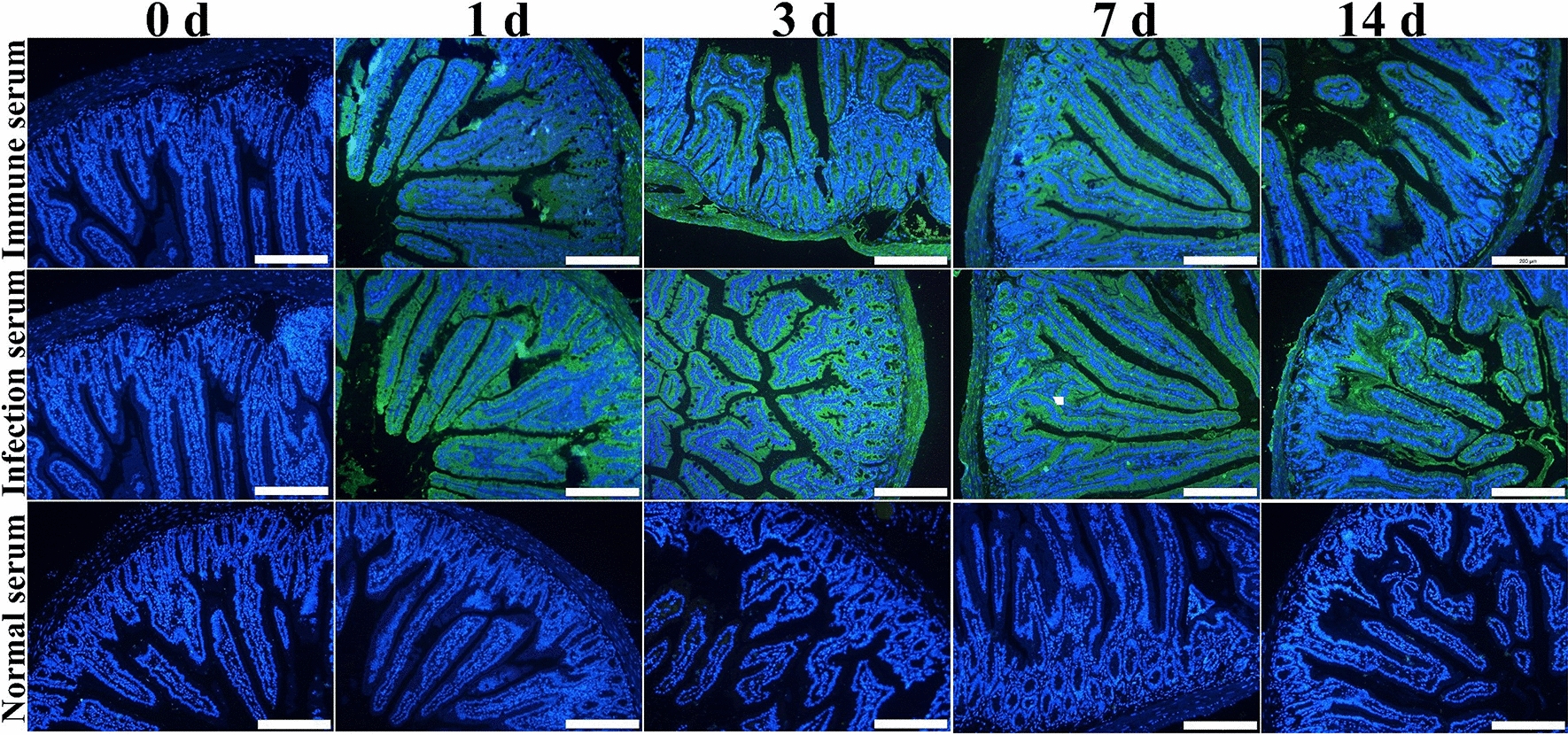
**Binding of native TsCTL with enteral epithelium from infected mice at various times after** ***T. spiralis*** **infection.** Intestinal sections from infected mice at different times after infection were probed with anti-rTsCTL immune serum and infection serum, and immunostaining on enteral epithelium was detected at 1, 3, 7 and 14 dpi. Cell nuclei were stained blue with DAPI. Scale bars: 200 μm.

The IFA results showed that after incubation with rTsCTL, green fluorescence on normal mouse enteral epithelium was detected by anti-rTsCTL serum and infection serum, but no immunostaining was observed by preimmune serum (Figure 10). When intestinal sections from infected mice at different times after infection were probed with anti-rTsCTL serum and infection serum, immunostaining of the enteral epithelium was detected at 1, 3, 7 and 14 dpi (Figure 11), demonstrating that natural TsCTL could be secreted and bind to the enteric epithelium at the intestinal stage of *T. spiralis* infection and that TsCTL might participate in larval invasion of the host’s gut mucosa.

### rTsCTL haemagglutination activity and sugar inhibition assays

**Figure 12:**
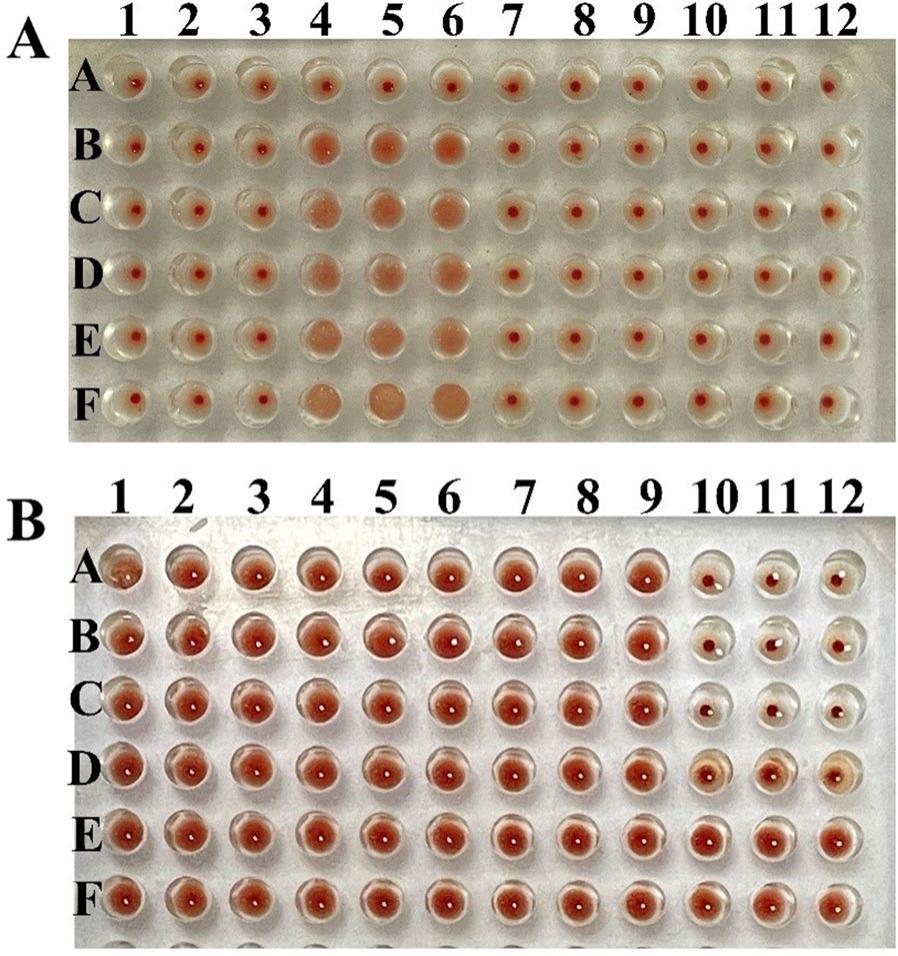
**rTsCTL haemagglutination activity and sugar inhibition. A:** Haemagglutination of mouse erythrocytes with various concentrations of rTsCTL. Lanes 1–3: TBS buffer + rTsCTL; Lanes 4–6: TBS buffer + CaCl_2_ + rTsCTL; Lanes 7–9: TBS buffer + CaCl_2_ + BSA; Lanes 10–12: TBS buffer + CaCl_2._ **A-F** Different concentrations of rTsCTL (0, 25, 50, 100, 200 and 400 μg/mL). **B:** Inhibition of diverse carbohydrates on rTsCTL haemagglutinating activity to mouse erythrocytes. Lane 1–3: lactose; Lane 4–6: sucrose; Lane 7–9: glucose; Lane 10–12: mannose. **A-F** are the sugar dilutions at different concentrations of 400, 200, 100, 50, 25 and 0 mM, respectively.

rTsCTL was used for the haemagglutination and sugar inhibition assays, and the results revealed that rTsCTL has a haemagglutinating function, which was Ca^2+^ dependent. The minimum concentration of rTsCTL agglutinating to mouse erythrocytes was 25 μg/mL (Figure 12A). In the sugar inhibition assay, mannose was the only carbohydrate that inhibited the agglutination of mouse erythrocytes by rTsCTL, and the minimum inhibitory dose of mannose was 100 mM (Figure 12B).

### rTsCTL promotion and mannose suppression of larval intrusion

**Figure 13:**
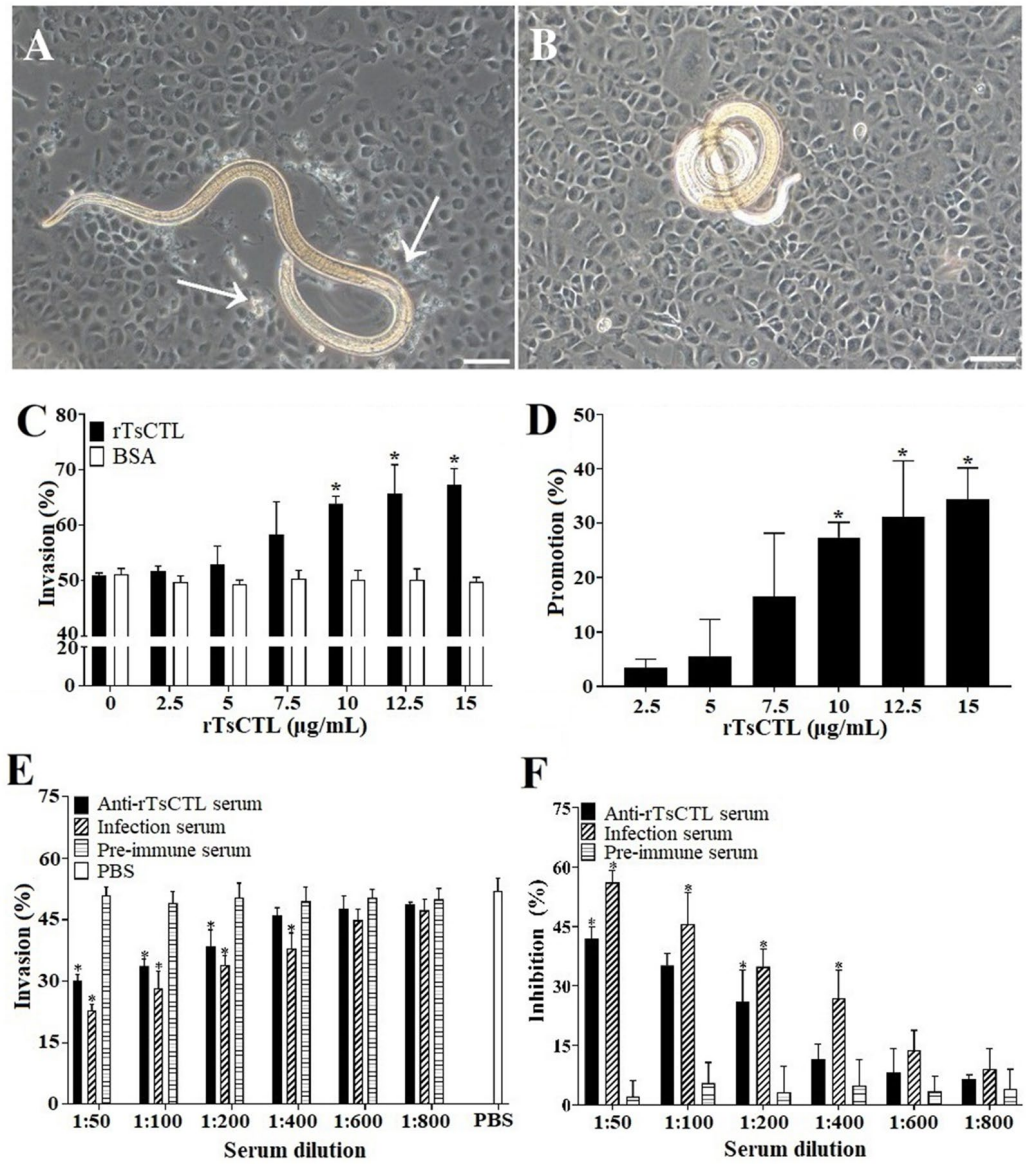
**Facilitation of rTsCTL on larval invasion of IECs.** The MLs were first activated into IILs using 5% porcine swine bile for 2 h at 37 °C and then added to the IEC monolayer, and larval invasion was observed under a microscope at 2 h after coculture. **A:** The invaded larva was mobile and migratory in the monolayer (the white arrow shows the migratory trace). **B:** Noninvaded larva was coiled on the IEC surface. **C** and **D:** rTsCTL accelerated the worm invasion of IECs. **E** and **F**: Inhibition of anti-rTsCTL antibodies on the IIL invasion of IECs. The results are expressed as the promotion or inhibition (%) normalized to the PBS control group. Scale bars: 100 μm. * *P* < 0.05 compared to the BSA and PBS control groups.

As shown in Figure 13A, invaded larvae left a clear migratory trace (white arrow), and noninvaded larvae were coiled on the cell monolayer surface (Figure 13B). When the medium was supplemented with rTsCTL and IILs were cultured in the medium for 2 h, rTsCTL obviously facilitated larval invasion. This acceleration was rTsCTL dose-dependent (*r* = 0.976, *P* < 0.01) and exhibited an increasing trend with increasing rTsCTL dose (*F* = 82.091, *P* < 0.01). However, BSA did not result in acceleration of larval invasion (Figures 13C, D). When various dilutions (1:50–1:200) of anti-rTsCTL serum were added to the medium and coincubated with IILs for 2 h, the inhibition of larval invasion of IECs was 41.99, 35.15 and 26.08%, respectively, compared to that of the PBS group (*χ*^*2*^_1:50_ = 10.242, *P* < 0.01; *χ*^*2*^_1:100_ = 7.126, *P* < 0.01; *χ*^*2*^_1:200_ = 3.911, *P* < 0.05). The inhibition was dose-dependent for anti-rTsCTL antibodies (*r* = 0.918, *P* < 0.05) and showed a declining trend with increasing serum dilution (*F* = 21.363, *P* < 0.05) (Figures 13E, F). Moreover, preimmune serum did not have any suppressive effects on larval penetration into IECs.

**Figure 14:**
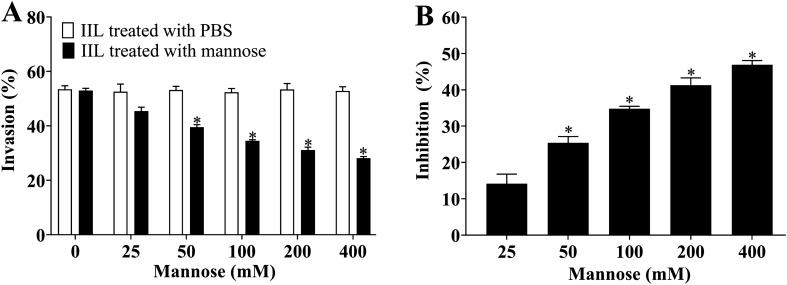
**Inhibition of larval invasion of IECs by mannose.** After IILs were first incubated with various doses of mannose (0–400 mM mannose) at 37 °C for 2 h, they were added to an IEC monolayer and cocultured for 2 h, and invaded larvae were examined under a microscope. **A** and **B**: Mannose (50–400 mM) evidently inhibited the larval invasion of IEC. *P < 0.05 compared to the PBS group.

After IILs were first incubated with various dilutions of mannose at 37 °C for 2 h, mannose-treated IIL was added to the IEC monolayer and cocultured for 2 h, and larval invasion was significantly suppressed. The suppression of larval invasion of IECs by 50–400 mM mannose was 25.39, 34.80, 41.27 and 46.88%, respectively, compared to that of the PBS group (χ^2^_50_ = 3.862, *P* < 0.05; χ^2^_100_ = 6.550,* P* < 0.05; χ^2^_200_ = 10.425,* P* < 0.001; χ^2^_400_ = 13.025, *P* < 0.0001) (Figure 14). The suppression had a correlation with the doses of mannose (*r* = 0.839, *P* < 0.05) and exhibited an elevating trend with the increase in mannose dose (*F* = 9.493,* P* < 0.05). The results showed that mannose evidently inhibited the IIL invasion of IECs and suggested that the preincubation of mannose and IILs might result in the binding of mannose with the TsCTL CRD, which reduced the interaction between the TsCTL CRD and IEC ligand, consequently inhibiting larval invasion.

**Figure 15:**
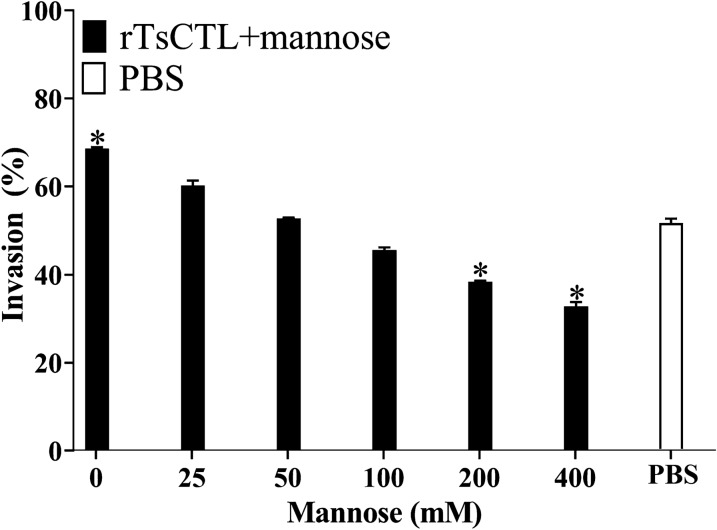
**Mannose reduced the rTsCTL facilitative effect on larval invasion of IECs.** rTsCTL (10 μg/mL) was first incubated with various doses of mannose (0–400 mM) for 2 h, and then, the IILs mixed with mannose-treated rTsCTL were added to the IEC monolayer and cocultured for 2 h. Mannose (200 and 400 mM) significantly reduced the rTsCTL facilitative act on larval invasion of IEC. *P < 0.05 compared to the PBS group.

To further verify whether mannose could block the facilitative effect of rTsCTL on larval penetration into IECs in vitro, we incubated 10 μg/mL rTsCTL at 37 °C for 2 h with various doses of mannose (0–400 mM), and then, the IILs mixed with mannose-treated rTsCTL were added to the IEC monolayer and cocultured for 2 h. The results showed that mannose (200 and 400 mM) evidently reduced the rTsCTL facilitative effect on larval invasion of IECs that compared to that of the PBS group without rTsCTL and mannose (χ^2^_200_ = 4.153, *P* < 0.05; χ^2^_400_ = 8.099, *P* < 0.05) (Figure 15).

## Discussion

Type C lectin (CTL) is a superfamily of more than 1000 proteins with one or more types of CLECT that bind carbohydrates in a Ca^2+^-dependent manner. The CLECT structure has a characteristic double-loop structure that is stabilized by two highly conserved disulphide bonds and a series of conserved hydrophobic and polar interactions, and it is most prominently characterized by the “WIGL” motif, which is highly conserved in CLECTs and participates in the formation of the hydrophobic core of CTL tertiary structures [65]. The CTL plays an important role in parasite infection. The CTL of parasitic helminths was first described in *Toxocara canis*, and CTLs secreted by *T. canis* can bind to ligands on the host cell surface. CTL promoted the penetration of *Schistosoma japonicum* into the host’s connective tissue [23]. The C-type lectin CpClec of *Cryptosporidium parvum* mediates the parasite invasion and infection of IECs [66].

In this study, a novel type C lectin domain protein (TsCTL, GenBank: KRY42391.1) was retrieved from the *T. spiralis* draft genome. TsCTL contains a signal peptide and a CLECT domain, and sequence alignment revealed a high amino acid sequence identity with CTLs of the 8 encapsulated *Trichinella* species/genotypes. Structural prediction showed that TsCTL had a CTL at residues 24–156. The results suggested that TsCTL may have similar functions in various species/genotypes of the genus *Trichinella*. The complete cDNA sequence of TsCTL was cloned into the pQE-80L plasmid and expressed in an *E. coli* expression system. Since the His tag had only six histidine residues, rTsCTL purification was convenient, and the His tag had little effect on the structure and property of rTsCTL, a single protein was obtained after purification using a Ni–NTA column [35, 67]. On Western blotting analysis, rTsCTL was identified by anti-rTsCTL serum and infection serum. Vaccination of mice with rTsCTL elicited a specific anti-rTsCTL IgG response, and the serum titre of specific anti-rTsCTL IgG was up to 1:10^5^, suggesting that rTsCTL had good immunogenicity.

qPCR results showed that TsCTL was transcribed in all the *T. spiralis* life-cycle phases, and the relative expression level of TsCTL in the 6 h IIL phase was obviously higher than that in the other stages. Western blotting analysis indicated that natural TsCTL in somatic and ES proteins of different worm phases was recognized by anti-rTsCTL serum. By immunofluorescence staining, native TsCTL was principally localized at the cuticle and stichosome and embryos of the female adults of this parasite. The stichosome consists of a series of stichocytes, mainly in the first half of the nematode. Each stichocyte possesses a single nucleus, and the cytoplasm contains characteristic distributed secretory granules that are highly antigenic. Additionally, each stichocyte contains a catheter leading to the oesophageal cavity that excretes the secreted proteins. Previous studies revealed that mannan-binding lectin (MBL) was located on the surface and internal organs of *T. spiralis* ML on histochemical staining. MBL bound to both *T. spiralis* ML crude extracts and ESA in a mannose-inhibiting manner [19]. The results suggested that TsCTL is a surface and secretory protein that is in direct contact with the host’s intestinal epithelium and might mediate IIL invasion of the enteric mucosa [30, 55].

Far-Western blot analysis can effectively screen for weak protein‒protein interactions from a crude mixture of proteins and has been successfully used to detect the binding of *T. spiralis* invasive proteins and IECs [28, 59]. The protein binding between TsCTL and IEC proteins was also ascertained in this study. Far-Western blot results indicated that there was a specific binding of rTsCTL and IEC proteins. In addition, the cellular localization of rTsCTL binding to IECs was examined by confocal microscopy, and the results demonstrated that the binding was mainly localized in the IEC nucleus and cytoplasm. The IFA results also showed that after incubation with rTsCTL, green fluorescence on normal mouse enteral epithelium was detected by anti-rTsCTL serum. When intestinal sections from infected mice at different times after infection were probed with anti-rTsCTL serum and infection serum, immunostaining on enteral epithelium was detected at 1, 3, 7 and 14 dpi, demonstrating that natural TsCTL was secreted and bound to enteral epithelium at the early intestinal stage of *T. spiralis* infection. Previous studies revealed that when the IILs were incubated with IEC, some *Trichinella* proteins produced by IILs passed into the IEC [42]. The CRD of *Entamoeba histolytica* Gal/GalNAC lectin has the capacity to bind to TLR2 and TLR4 in human colonic cells, activate their signalling pathway and facilitate trophozoite adhesion to the cells [68]. The results further indicated that there is an interaction between TsCTL and IECs, and TsCTL might participate in larval invasion of the host’s gut epithelia [13]. However, it is necessary to verify which kinds of IEC proteins bind with TsCTL in further studies by using immunoprecipitation and mass spectrometry [16].

The haemagglutination activity of rTsCTL was also identified in the current study. The results showed that rTsCTL has the function of haemagglutinating murine mouse erythrocytes and that its haemagglutination activities were Ca^2+^ dependent. In the sugar inhibition assay, mannose out of four carbohydrates was the only carbohydrate that could inhibit the agglutination of mouse erythrocytes by rTsCTL. Mannose, a hydroxyl-differential isoform at the C-2 position, is a monosaccharide involved in glycosylation modification that can specifically bind to CLECTs of some CTLs and inhibit the ability of CTLs to act with compound oligosaccharides [69]. The specific binding of rTsCTL to mannose was most likely related to their structure [19].

The results of the in vitro larval invasion test showed that rTsCTL facilitated the larval invasion of IECs, whereas anti-rTsCTL antibodies inhibited larval invasion; the facilitation or inhibition was dose-dependently related to the rTsCTL and anti-rTsCTL antibodies. This facilitation may be involved in the binding of rTsCTL with IECs [51]. Anti-rTsCTL antibody inhibition of larval invasion of IECs was likely due to cap-like immune complex formation of TsCTL and anti-TsCTL antibodies at the worm anterior, which blocked larval direct contact with gut epithelia and impeded worm invasion [15]. Moreover, after IILs were incubated with various dilutions of mannose, larval invasion was significantly suppressed, and the suppression exhibited an elevating trend with increasing mannose dose. The inhibition of mannose on larval invasion of IECs is likely because TsCTL was localized at the epicuticle of IILs, and the binding of mannose and TsCTL CRD on the surface of IILs competitively suppressed the binding of TsCTL to the ligand of IECs, which might block the interaction and binding of rTsCTL with IEC, impeding the larval invasion of IECs. A previous study showed that the binding of the *C. parvum* C-type lectin CpClec and IECs was specifically inhibited by sulphated glycosaminoglycans, and *C. parvum* attachment to and infection of HCT-8 cells were also inhibited by glycosaminoglycans [66]. *T. gondii* invasion into host cells was promoted by this parasite C-type lectin-CD209 interaction and inhibited by ligand mimicking-oligosaccharides and the anti-CD209 antibody. These oligosaccharides also reduced parasite burden, host spreading and mortality associated with *T. gondii* infection [70]. The detailed in vivo function of TsCTL needs to be verified by animal infection experimentation in further studies. The results suggested that mannose is likely used as an adjuvant agent for anti-*Trichinella* drugs and vaccines to block larval invasion at the early stage of *Trichinella* exposure and infection.

In conclusion, TsCTL was highly expressed at the IIL stages of the *T. spiralis* lifecycle and localized to the cuticle, stichosome and embryos of female adults. TsCTL is a surface and secretory protein that binds specifically to IECs and the gut epithelium, and the binding sites are localized in the nucleus and cytoplasm of IECs. rTsCTL had haemagglutination activities against erythrocytes, and mannose inhibited the haemagglutinating function of rTsCTL. rTsCTL could promote the larval invasion of IECs, while anti-rTsCTL serum and mannose inhibited the larval invasion of IECs in a dose-dependent manner. These findings demonstrated that TsCTL plays a principal role in *T. spiralis* invasion of the gut mucosa and might be a candidate target for vaccines against *T. spiralis* invasion and early infection.

## Abbreviations

AWs: adult worms
DAB: 3, 3'-Diaminobenzidine tetrahydrochloride
DAPI: 4', 6-Diamidino-2-phenylindole
ESA: excretory/secretory antigens
FBS: foetal bovine serum
HRP: horseradish peroxidase
IECs: intestinal epithelial cells
IILs: intestinal infective larvae
IFA: immunofluorescence assay
IPTG: isopropyl β-d-1-thiogalactopyranoside
MLs: muscle larvae
NBLs: newborn larvae
NC: nitrocellulose
OD: optical density
PBS: phosphate-buffered saline
TBST: Tris-buffered saline containing Tween
TsCTL: *Trichinella spiralis* Type C lectin
